# Supplementary material for: Differences in Maternal Immunoglobulins within Mother’s Own Breast Milk and Donor Breast Milk and across Digestion in Preterm Infants
Source: Nutrients. 2019 Apr 24;11(4):920. doi: 10.3390/nu11040920 (PMC6521323; doi:10.3390/nu11040920)
Supplement: Supplementary file 1 [file nutrients-11-00920-s001.pdf]

Table S1. Statistical results (p-values) for Student's t-tests to compare pH, protein concentration and antibody concentration between samples from 8–9 days (n = 20) and 21–22 days (n = 16) of postnatal age for each sample type (feed, gastric and stools) (separated days of postnatal age). MBM, mother's own breast milk; DBM, donor breast milk.

Measurements	MBM		DBM		
	Feed	Gastric	Feed	Gastric	Stool
pH	0.13	0.16	0.29	0.19	
Protein concentration, mg/mL	0.001***	0.36	0.22	0.47	0.24
Total IgA, µg/mL	0.19	0.80	0.062	0.14	0.33
SIgA, µg/mL	0.089	0.22	0.44	0.61	0.28
Total IgM, µg/mL	0.070	0.058	0.83	0.11	0.37
IgG, µg/mL	0.060	0.53	0.81	0.24	0.69

Table S2. Statistical results (p-values) for Student's t-tests comparing antibody concentration between infants that received antibiotics (n = 5 at 8–9 days and n = 5 at 21–22 days of postnatal age) and infants that did not receive antibiotics (n = 15 at 8–9 days and n = 8 at 21–22 days of postnatal age) in gastric and stool samples (combined time of postnatal age). MBM, mother's own breast milk; DBM, donor breast milk.

Samples	SIgA	Total IgA	Total IgM	IgG
Gastric MBM	0.086	0.75	0.20	0.85
Gastric DBM	0.23	0.12	0.75	0.13
Stool	0.73	0.21	0.51	0.33

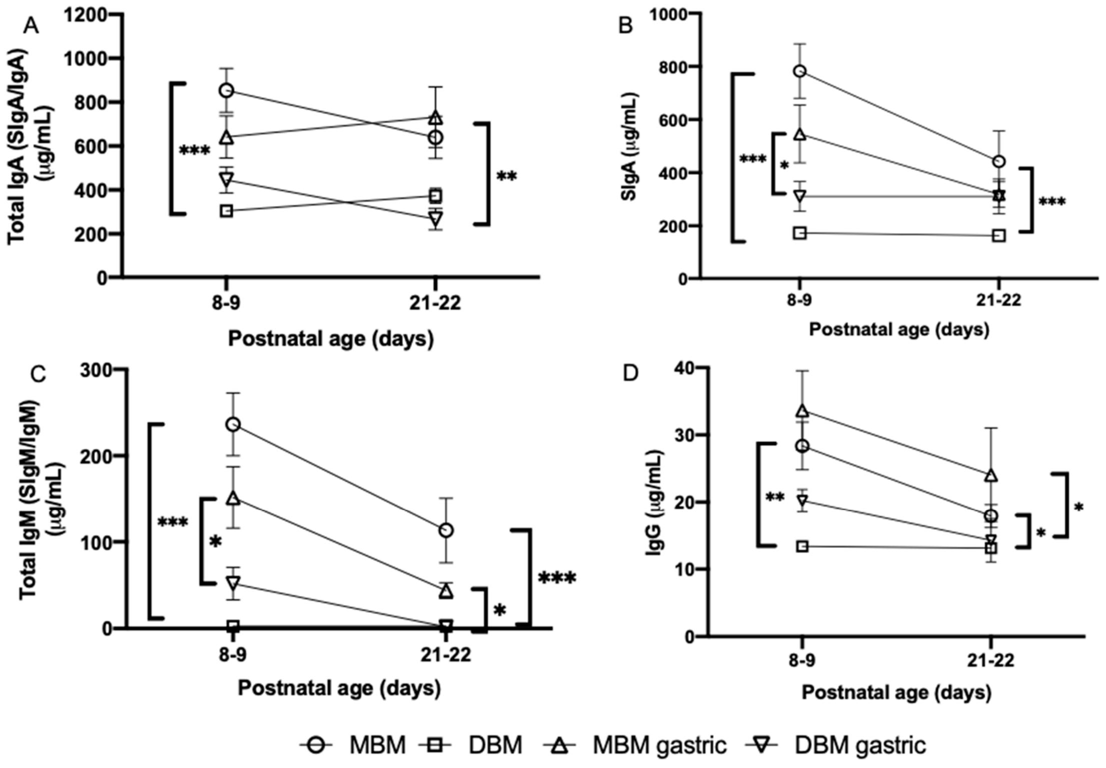

Figure S1. Immunoglobulin concentrations in milk and gastric contents at 1-hour postprandial time from 20 preterm infants (26–36 weeks of gestational age (GA)) fed mother's own breast milk (MBM) and donor breast milk (DBM) at 8–9 days and 21–22 days of postnatal age. Concentration of (**A**) total IgA (SIgA/IgA), (**B**) secretory IgA (SIgA), (**C**) total IgM (SIgM/IgM) and (**D**) IgG in milk and gastric samples. Values are mean ± SEM, $n = 20$ for 8–9 days for MBM and DBM and $n = 16$ for 21–22 days of postnatal age for MBM and DBM). Asterisks show statistically significant differences between variables (*** $p < 0.001$; ** $p < 0.01$; * $p < 0.05$) using the Wilcoxon matched-pairs signed-rank test.

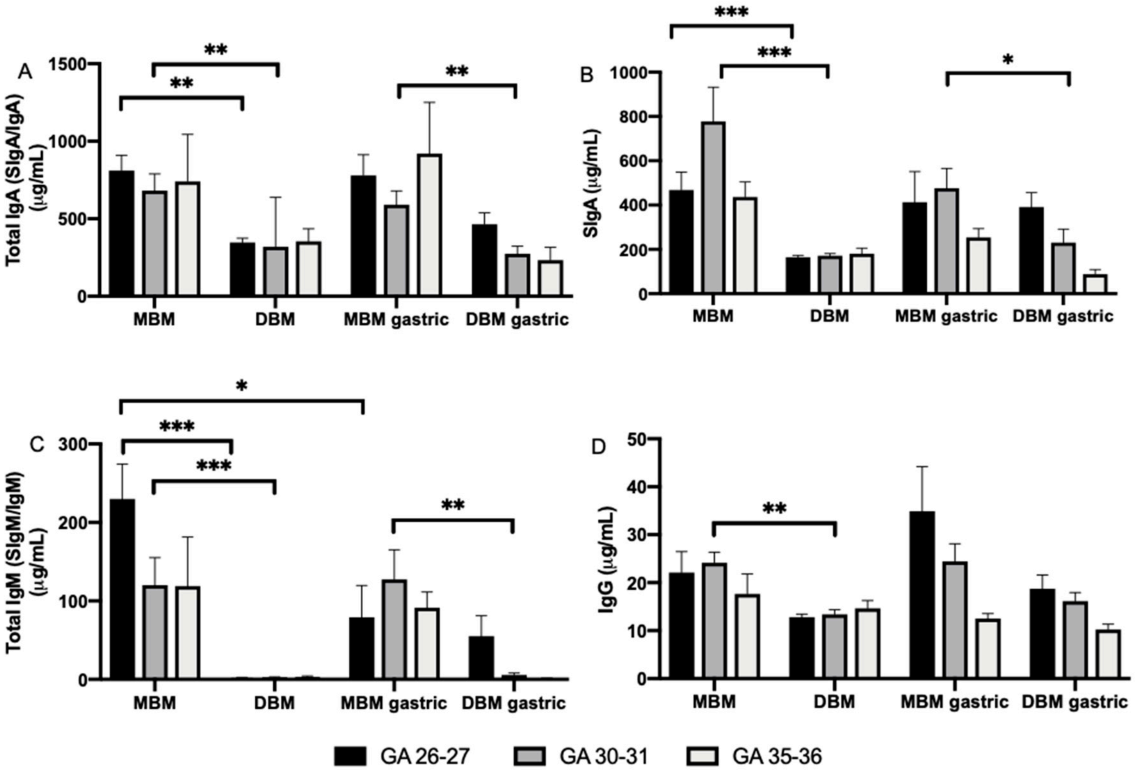

Figure S2. Immunoglobulin concentrations in milk and gastric contents at 1-hour postprandial time from 3 gestational age (GA) groups of preterm infants (26–27 weeks of GA, 30–31 weeks of GA, 35–36 weeks of GA) fed mother's own breast milk (MBM) and donor breast milk (DBM). Concentration of (**A**) total IgA (SIgA/IgA), (**B**) secretory IgA (SIgA), (**C**) total IgM (SIgM/IgM) and (**D**) IgG in milk and gastric samples. Values are mean ± SEM, $n = 8$ for G 26–27 ($n = 4$ for 8–9 and $n = 4$ for 21–22 days of postnatal age for MBM and DBM); $n = 8$ for G 30–31 ($n = 4$ for 8–9 days and $n = 4$ for 21–22 days of postnatal age for MBM and DBM), $n = 4$ for G 35–36 (8–9 days of postnatal age). Asterisks show statistically significant differences between variables (*** $p < 0.001$; ** $p < 0.01$, * $p < 0.05$) using the Wilcoxon matched-pairs signed-rank test.

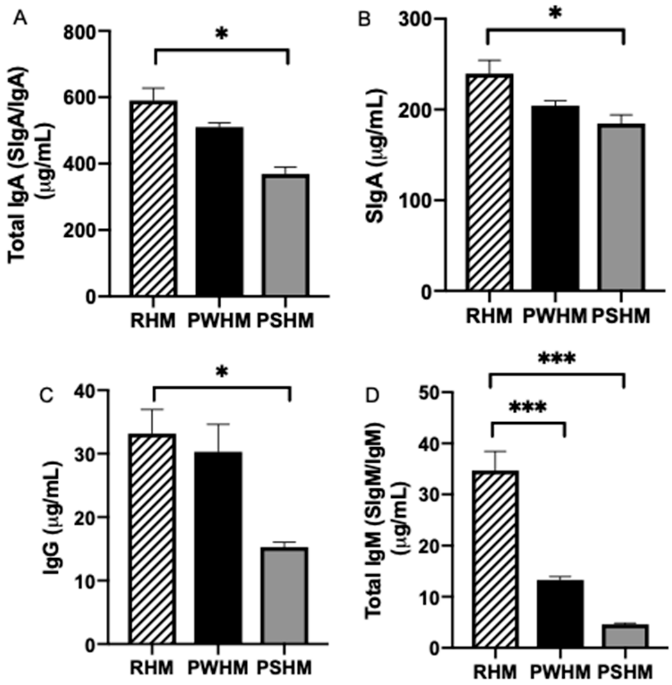

Figure S3. Comparison of immunoglobulin concentration between raw human milk (RHM) and pasteurized whole human milk (PWHM) or pasteurized skimmed human milk (PSHM) (n = 3). Concentration of (A) total IgA (SIgA/IgA), (B) SIgA, (C) total IgM (SIgM/IgM) and (D) IgG in mother's milk. Milk samples were from one mother who delivered one term infants with 38 weeks of gestational age and 12 days of postnatal age. Asterisks show statistically significant differences between variables (***$p < 0.001$; *$p < 0.05$) using One-way ANOVA followed by Dunnett's multiple comparisons test.
